# Correlation of T-regulatory Cells and Iron Status in β-Thalassemia Major Patients

**DOI:** 10.7759/cureus.35084

**Published:** 2023-02-16

**Authors:** Farah Choudhary, Poonam Rani, Mrinalini Kotru, Sunil Gomber, Pooja Dewan, Richa Gupta, Meera Sikka, Shilpi More

**Affiliations:** 1 Pathology, University College of Medical Sciences, Delhi, IND; 2 Pediatrics/Oncology, University College of Medical Sciences, Delhi, IND; 3 Pediatrics, University College of Medical Sciences, Delhi, IND; 4 Pathology, Employees' State Insurance Corporation (ESIC) Medical College and Hospital, Faridabad, IND

**Keywords:** β -thalassemia major, cd8, cd4, t-regulatory cells, cell mediated immunity, iron

## Abstract

Background

The increased risk of infections in transfusion-dependent β-thalassemia major (TDT) patients is mainly due to underlying immune dysfunction; however, its cause is largely unidentified. There is sufficient evidence to suggest immune changes due to iron deficiency; however, similar studies demonstrating the effects of iron excess on immune cells in these cases are limited.

Aim and objectives

To analyze the correlation between T-regulatory cells and iron stores in β-thalassemia major patients.

Methods

In this study, 20 β-thalassemia major cases and 20 healthy controls were studied for complete hemogram, iron profile, and flow cytometric immunophenotyping for CD3+, CD4+, CD8+, and T-regulatory cells markers (CD4+CD25+ and CD4+CD25+FOXP3+).

Result

Significantly higher levels of serum iron, ferritin, transferrin saturation, and CD4+ cell percentage were observed in cases than in controls. In 70% of cases with serum ferritin cut-off levels of less than 1000 µg/L, the T-regulatory cell marker CD4+CD25+ and serum ferritin revealed a significant moderate positive correlation (p=0.031, r=0.627). These same 70% cases also demonstrated a moderately significant positive correlation between serum iron and absolute lymphocyte count (r=0.529, p=0.042).

Conclusion

The results suggest that serum ferritin in excess amounts can increase T-regulatory cells, which may further alter the immune status of TDT patients; however, the absence of such a correlation in cases with serum ferritin of more than 1000 µg/L remains unanswered. It is important to understand immune system alterations as this will help provide new modalities for managing thalassemia patients in the form of immunoregulatory therapies.

## Introduction

Hemoglobinopathies comprise a major health burden in India and the world. Of these, transfusion-dependent β-thalassemia major is the foremost group. A wide variation in prevalence is noted, ranging from 0.94% in central India up to 13.20% in eastern India [1]. These patients suffer from severe anemia necessitating regular blood transfusions, which expose them to various blood antigens, thereby predisposing them to alloimmunization as well as the risk of iron overload. Infections and complications significantly contribute to morbidity and mortality in β-thalassemia major patients. Besides the increased risk of transfusion-transmitted infections, a concurrent immunodeficient state makes them more susceptible to acquiring infectious diseases. The etiopathogenesis of recurrent infections in these patients is not clear. Whether the immunity is altered because of repeated antigenic exposure, thalassemia itself, or iron overload is a matter of debate. The repeated blood transfusions in these patients lead to constant antigenic stimulation; however, as expected, most of the patients do not have alloimmunization. This immune tolerance is possibly due to increased T-regulatory cells [2]. Transfusion-dependent thalassemia major patients have also been observed to be prone to repeated infections, probably because of the alteration in T-regulatory cell numbers [3].

Limited studies explore the T-regulatory cells in β-thalassemia major cases, and even fewer focus on the association between iron overload and cell-mediated immunity. So, this study was conducted with the hypothesis that excess iron might affect the T-regulatory cells and other cell-mediated immune cells such as CD3, CD4, and CD8.

## Materials and methods

This case-control study was conducted in the Department of Pathology and Pediatrics where 20 diagnosed β-thalassemia major cases and 20 healthy controls (+/- 2 years of age) were studied. A small number of subjects was selected due to resource limitations in terms of flow cytometry antibodies. The β-thalassemia major cases placed in excluded criteria were patients with a history of diabetes mellitus, HIV, HCV, or immunosuppressants due to the established significant effects of these entities on T-regulatory cells. Written informed consent/assent was taken from all the patients and controls. The study received approval from the Institutional Ethics Committee-Human Research (IEC-HR).

The blood samples for the hematology work-up, iron studies, and immunophenotyping were taken before the scheduled blood transfusion. The EDTA (ethylenediamine tetraacetic acid) sample was subjected to analysis on an automated hematology analyzer (Mindray BC-6800, Mindray Medical International Ltd., Shenzhen, China) for a complete hemogram.

The complete iron profile was done, which included the estimation of serum iron (SI), total iron binding capacity (TIBC), percentage transferrin saturation (TSAT), and serum ferritin (SF). The venous blood sample was collected in an iron-free tube and allowed to clot undisturbed for two hours at 37°C. When clot retraction was complete, the sample was centrifuged, the serum was removed by an iron-free pipette into an iron-free tube, and 6 mL was stored in an Eppendorf tube at -20°C. The serum iron was measured according to ICSH (International Council for Standardization in Haematology) guidelines [4]. The values of serum iron considered normal in our laboratory are 60-170 μg/dl. The normal value of TIBC is 250-400 μg/dL, and that of TSAT is 16-50%. The serum ferritin was assessed using a commercially available ELISA (enzyme-linked immunosorbent assay) kit (Calbiotech Inc., El Cajon, CA). The absorbance was read on an ELISA reader (Bio-Rad Laboratories Inc., Hercules, CA) at 450 nm within 15 min after adding the stop solution. The value considered normal for serum ferritin is 20-300 µg/L for men and 10-200 µg/L for women. The alloimmunization status of all the cases was evaluated using a 3-cell panel kit from Bio-Rad Laboratories for Kell, Duffy, and E antigens.

Flow cytometric analysis

Immunophenotyping was done on an EDTA sample on the Beckman Coulter FC-500, a 5-color flow cytometer (Beckman Coulter Inc, Brea, CA), by the stain-lyse-wash method to first identify T cells and categorize them into CD4 and CD8 T cells. Then, two tubes were taken to identify T-regulatory cells out of the CD4+ T population. One tube was stained with a cocktail containing CD45, CD4, CD8, and CD3 in 70 μl of blood. In another tube, 70 μl blood was stained with CD45, CD4, CD3, CD25, and FOXP3, according to the panel below (Table 1).

**Table 1:** Antibody panel for flowcytometric analysis FITC: Fluorescein isothiocyanate, PE: Phycoerythrin, ECD: Phycoerythrin-Texas Red conjugate (energy-coupled dye), PC5: Phycoerythrin-cyanine5 conjugate, PC7:Phycoerythrin-cyanine7 conjugate

Fluorescence indicators	Tube 1		Tube 2	
	Cluster of differentiation (CD Markers)	Clone	Cluster of differentiation (CD Markers)	Clone
FITC	CD45	J33(IgG1 Mouse)	CD45	J33(IgG1 Mouse)
PE	CD4	13B8.2(IgG1 Mouse)	Anti-FOXP3	259D (IgG1Mouse)
ECD	CD8	B9.11(IgG1 Mouse)	CD4	SFCI12T4D11(IgF1Mouse)
PC5	CD3	UCHT1(IgG1Mouse)	CD3	UCHT1(IgG1 Mouse)
PC7	Nil	Nil	CD25	B1.49.9(IgG2a Mouse)

Gating strategy

**Figure 1:**
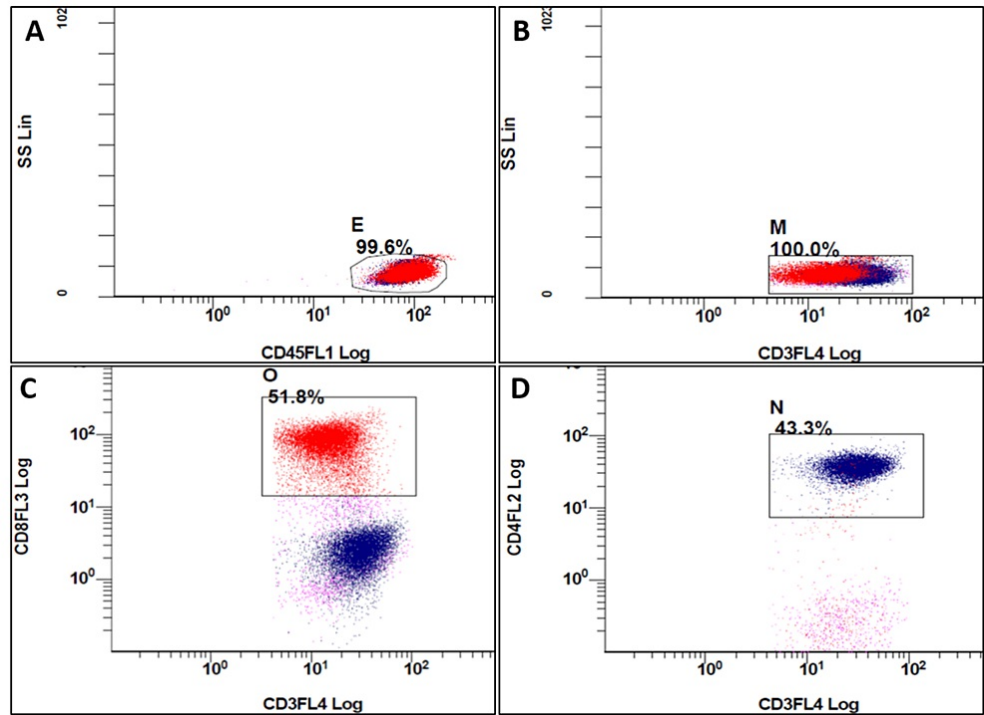
Steps for identifying CD4 and CD8 T-lymphocytes in Tube 1. Co-positive cells for CD4/CD3 and CD8/CD3 were taken as CD4 and CD8 T-lymphocytes respectively. Figure 1A: Plot CD45 versus side scatter (SS), clean lymphocyte population with CD45 high and low side scatter was selected. Figure 1B: The lymphocyte gating on CD3 versus side scatter (SS) to get CD3+ T-lymphocytes (red and blue). Figure 1C: The cells co-positive for CD3 and CD8 (red) were considered CD8+ T-lymphocytes. Figure 1D: The cells co-positive for CD3 and CD4 (Blue) were taken as CD4+ T-lymphocytes (T-Helper cells).

The viability gating was done on side scatter (SSC) versus CD45 to exclude the debris. Then a sequential gate was put on SSC versus CD3 to include CD3-positive T-lymphocytes. Low-side scatter cells were selected to exclude monocytes. Further gating was done on CD4 versus CD3 to isolate a pure population of T-helper cells. The steps for identifying CD4 and CD8 T-lymphocytes in Tube 1 have been shown in Figure 1A-1D.

T-regulatory cell characterization

**Figure 2:**
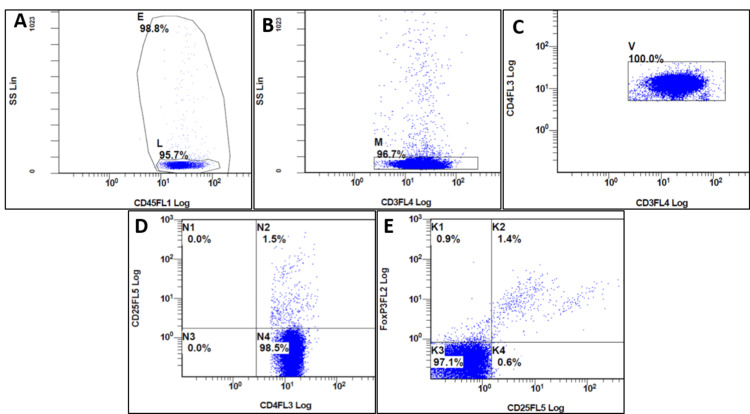
Steps (2A-2E) for identifying T-regulatory cells (T-regs). The T-regs were identified in N2 quadrant and K2 quadrant as CD4+/CD25+ and CD4+/CD25+/FOXP3+ respectively on CD4 T-lymphocyte gating. Figure 2A: The lymphocyte population was selected by viability gating done on side scatter (SS) versus CD45 plot to exclude the debris. Figure 2B: Then a sequential gate was put on SS versus CD3 plot to include CD 3 positive T-lymphocytes. Low-side scatter cells were selected to exclude monocytes. Figure 2C: The CD4 versus CD3 plot, cells co-positive for both the markers were selected to isolate a pure population of T-helper cells. Figure 2D: The T-regulatory cells were identified on the CD4/CD25 plot (N2 quadrant). Figure 2E: Another plot to identify T-regulatory cells with CD4/CD25/FOXP3 antibodies (K2 quadrant). The CD4+ cells co-expressing CD25 and FOXP3 were taken as T-regulatory cells.

In the second tube, the gating was done on CD4 versus CD3 to isolate T-helper cells, and then T-regs were identified on CD4/CD25 and CD4/CD25/FOXP3 plots. The T-regulatory cells were quantified using CD4+/CD25+ and CD4+/CD25+/FOXP3+ expression on CD4+T lymphocytes. The steps for identifying T-regulatory cells in the second tube have been demonstrated in Figure 2A-2E.

Treatment guidelines for cases

All the cases received a regular blood transfusion and were managed as per the latest TIF (Thalassemia International Federation) guidelines for the management of iron overload in patients with hemoglobinopathies [5]. The chelation was started for them if their serum ferritin levels increased above 1000µg/L or when they have received up to 10 blood transfusions, whichever was earlier. The chelation therapy regime was deferiprone (DFP) of 75-100mg/kg/day orally, deferasirox (DFX) of 20-40mg/kg/day orally, or their combination depending on iron overload. The hepatic and cardiac iron overload was assessed by magnetic resonance-based methods standardized and regularly calibrated to specifically measure iron load. All the cases were also periodically investigated to look for endocrinological involvement. 

Statistical analysis

The collected data was transformed into variables, coded, and entered in Microsoft Excel (Microsoft Corp., Redmond WA). Data were analyzed and statistically evaluated using the SPSS for Windows v. 20 (IBM Corp., Armonk, NY). Quantitative data were expressed in mean ± standard deviation or median with interquartile range and differences between two comparable groups were tested by Student’s t-test (unpaired) or Mann Whitney ‘U’ test while qualitative data were expressed in percentage and statistical differences between the proportions were tested by chi-square test or Fisher’s exact test. Pearson correlation coefficient was used to see the correlation between two quantitative variables. A p-value less than 0.05 was considered statistically significant.

## Results

The total number of cases and controls was 40. The age of cases ranged from 3-24 years with mean ± SD of 15.75±5.01 years. The age of the control group ranged from 5-22 years with a mean ± SD of 16.20±4.37 years. All the cases demonstrated significantly higher serum ferritin levels than controls. The 14 (70%) cases who demonstrated serum ferritin levels below 1000 µg/L were categorized as a non-iron overload group. On the other hand, 30% of cases with serum ferritin levels above 1000 µg/L were categorized as iron overload group. The age of the iron overload cases ranged from 12 to 23 years with a mean ± SD of 16.00±4.51 years. The age of the non-iron overload cases ranged from 3-24 years with a mean ± SD of 15.64±5.37 years. No significant difference in hematological parameters was noted between iron overload and non-iron overload cases. None of the cases in our study developed alloantibodies to Kell, Duffy, or E antigen. None of the cases underwent splenectomy before or during the study.

Hematological profile

There were significant differences in hemoglobin, hematocrit, red blood cell (RBC) count, mean corpuscular hemoglobin (MCH), reticulocyte count, erythrocyte sedimentation rate (ESR), and platelet count values of cases and controls as the p-value was less than 0.05. No significant difference in hematological parameters between Iron overload and non-iron overload cases was noted. None of the cases in our study developed alloantibodies to Kell, Duffy, or E antigen (Table 2).

**Table 2:** Comparison of hematological parameters between cases and controls Hb: hemoglobin; HCT: hematocrit; RBC: red blood cell count; MCV: mean corpuscular volume; MCH: mean corpuscular hemoglobin; MCHC: mean corpuscular hemoglobin concentration; ESR: erythrocyte sedimentation rate; TLC: total Leucocyte count; ALC: absolute lymphocyte count *p-value <0.05 considered significant

Parameters	Groups		p-value
	Cases (Mean ± SD)	Controls (Mean ± SD)	
Hb(g/dL)	8.97±1.44	13.62±2.74	<0.001 *
HCT (%)	27.47±3.78	40.25±8.11	<0.001*
RBC(x1012/L)	3.18±0.35	4.52±0.91	<0.001*
MCV (fL)	84.03±8.45	87.68±12.85	0.55
MCH (pg)	27.81±3.01	30.24±3.95	0.01*
MCHC (g/dL)	32.99±0.79	32.95±2.46	0.71
Reticulocyte (%)	0.40±0.63	1.21±1.39	0.02*
ESR (mm/hr)	25.80±14.77	9.20±19.39	<0.001*
TLC (×109/L)	7.68±2.99	7.01±1.61	0.34
Lymphocyte	27.40±6.28	31.25±11.49	0.32
ALC (/mm3)	2056.75±831.59	2161.55±827.13	0.71
Platelets (×109/L)	323.05±148.94	221.50±59.58	0.02*

Immune alteration in β-thalassemia major cases and controls

The T-regulatory cell markers CD4+CD25+ and CD4+CD25+FOXP3+ were found to have a strong correlation and were in concordance with each other (r=0.92) (p<0.001), suggesting that both of these markers can be used interchangeably for identification of T-regs. The percentage of CD4+ cells was found to be significantly increased in cases than in controls (p=0.03). No significant difference was noted in T-regs, CD8%, CD4: CD8 ratio, and T-lymphocytes of cases and controls (Table 3).

**Table 3:** Comparison of cell-mediated immune cells among cases and controls *p-Value <0.05 considered significant

Cell-mediated immune cells	Groups		p-value
	Cases (Mean ±SD)	Controls (Mean ± SD)	
CD4%	51.81±7.19	46.08±15.07	0.03*
CD8%	32.01±8.90	30.79±9.31	0.95
CD4:CD8 Ratio	1.76±0.68	3.65±10.21	0.09
T lymphocytes %	70.43±5.88	74.35±9.50	0.12
CD4 +CD25+	1.64±1.32	2.01±1.24	0.15
CD4+CD25+ FOXP3+	1.47±1.09	1.74±1.01	0.20

Iron profile of cases and controls

The serum iron, serum ferritin, and transferrin saturation (TSAT) were significantly higher in cases than in controls (p<0.05) however, TIBC did not show any difference (Table 4).

**Table 4:** Comparison of iron-related parameters between cases and controls SI: serum iron; TIBC: total iron binding capacity; TSAT: transferrin saturation *p-value <0.05 considered significant

Parameters	Group		p-value
	Cases (n=20)	Controls (n=20)	
SI, µg/dL (Mean ± SD)	236.55±51.26	87.10±40.44	<0.001*
TIBC, µg/dL (Mean ± SD)	343.45±103.35	363.95±51.54	0.16
TSAT, % (Mean ± SD)	72.60±18.38	24.75±12.76	<0.001*
Serum Ferritin (SF), µg/L (Mean± SD)	1134.55±1075.95	100.20±144.17	<0.001*

Iron profile of iron overload and non-iron overload groups

The mean serum ferritin± SD among iron overload cases was 2533.00 ± 981.90 while it was 537.21±189.29 for non-iron overload cases. Among the two groups of cases, significantly high serum ferritin levels were seen in iron overload cases as compared to non-iron overload (p=0.004). No significant difference was noted in SI, TIBC, and TSAT between the two groups of cases. In iron overload cases, serum ferritin showed a moderate positive correlation with CD4+CD25+ (r=0.543) and a weak negative correlation with the percentage of CD8+ cells (r=-0.441), which was not statistically significant.

Immune profile of iron overload and non-iron overload groups

The percentage of T- lymphocytes was significantly higher in iron overload cases than in non-iron overload cases (p=0.005). In non-iron overload cases, serum iron demonstrated a significant moderate positive correlation with absolute lymphocyte count (r=0.529, p=0.042). Similarly, in these patients, serum ferritin showed a significant moderate positive correlation with T-regulatory cell marker CD4+CD25+ (r=0.627, p=0.031).In contrast, the iron overload cases did not show any correlation with CD4, CD8, CD4:CD8 ratio, or T-regulatory cells.

Immune profile of healthy controls

The percentage of CD8+ cells demonstrated a significant negative correlation with serum ferritin (r= -0.607, p-value= 0.005) while a significant weak positive correlation with TIBC (r=0.447, p-value=0.048). The T-regulatory cell markers CD4+CD25+ and CD4+CD25+FOXP3+ demonstrated a significant moderate positive correlation with serum iron and transferrin saturation (Table 5).

**Table 5:** Correlation of iron parameters with cell-mediated immunity in healthy controls TIBC: total iron binding capacity; TSAT: transferrin saturation *p-value <0.05 considered significant

Cell-mediated immune cells	Correlation (r and p-value)	Serum Iron	TIBC	TSAT	Serum ferritin
CD4%	r value	0.129	-0.159	0.141	0.022
	p value	0.589	0.502	0.552	0.927
CD8%	r value	0.020	0.447*	-0.221	-0.607*
	p value	0.935	0.048*	0.349	0.005*
CD4:CD8	r value	0.137	-0.338	0.226	0.211
	p value	0.566	0.145	0.338	0.373
CD4+CD3+ (T-helper cells)	r value	-0.197	-0.173	-0.132	0.265
	p value	0.405	0.466	0.578	0.259
CD4+CD25+	r value	0.650*	-0.165	0.599*	0.252
	p value	0.002*	0.486	0.005*	0.284
CD4+CD25+FOXP3+	r value	0.522*	-0.106	0.484*	0.020
	p value	0.018*	0.656	0.031*	0.933
T-lymphocytes	r value	-0.126	-0.200	-0.005	0.247
	p value	0.598	0.398	0.985	0.294

Two-year follow-up results

None of the cases demonstrated hepatic, cardiac, or endocrinological iron overload at the start of the study, and all of them received single oral chelator DFX (20-40mg/kg/day). Two cases were lost to follow-up. Currently, only 33% of cases were found to be managed by oral chelator monotherapy, either DFP or DFX. The rest of the cases were on combination chelation regimes of DFP+DFX (50%), DFP+DFO (11%), or DFP+DFX+DFO (5%). Initially, serum ferritin levels above 1000 µg/L were demonstrated in only 30% of cases, which increased to 94.4% of cases after two years. The patients were categorized as normal, mild, moderate, or severe iron overload as per the following criteria. The T2-weighted MRI (T2 MRI) criteria for cardiac iron overload were as follows: normal >20 milliseconds (ms), mild: 14-20 ms, moderate: 10-14 ms, and severe <10 ms. The T2 MRI criteria for hepatic iron overload were as follows: normal >6.3 milliseconds (ms), mild: 2.8-6.3 ms, moderate: 1.4-2.7 ms, severe <1.4 ms [6].

In our study, the recent T2 MRI for a hepatic iron overload of cases demonstrated normal hepatic iron in 27.7% of cases, mild overload in 50% of cases, moderate in 16.6%, and severe in 5.5% of cases. The T2 MRI for cardiac iron overload demonstrated normal iron in 72.2% of cases, mild in 22% and moderate in 5.5% of cases. None of the cases demonstrated severe iron overload. 

None of the cases in our study demonstrated any infections such as HIV, HBV, HCV, or any other co-morbidities during the two-year follow-up period. However, recent workup revealed hepatic and cardiac iron overload in 70% and 27% of cases respectively. No endocrinological involvement was seen in our cases.

## Discussion

Transfusion-dependent thalassemia major patients face many problems, such as severe anemia due to marked hemolysis combined with ineffective erythropoiesis. Repeated transfusions are given to combat anemia, bringing the patient towards iron overload in vital organs and leading to hepatic, cardiac, and endocrinological dysfunction. Simultaneously, repeated transfusions enhance the risk of infections, whether due to transfusion, immune alterations by repeated antigenic exposure, iron overload, or the effect of chelators. All of our cases were anemic, as we collected the blood sample before the stipulated date for transfusion. There was a statistically significant difference in the hemoglobin of cases due to ineffective erythropoiesis compared with controls.

The absolute lymphocyte count (ALC) showed no significant difference between our cases and controls. However, Gharagozloo et al. [7] noted increased ALC in cases as compared to controls. This difference in ALC could be due to a larger sample size, high serum ferritin for six months in all cases, and varying doses of desferrioxamine mentioned in that study. More detailed studies are required for a better comparison of results. The cases and controls in our study did not reveal any significant difference in T-lymphocytes%, CD8%, or the CD4:CD8 ratio, while CD4% was significantly higher in cases than controls. This may be due to repeated antigenic stimulation because of blood transfusion, which increases the repertoire of memory T cells. Our results were in concordance with those of Gharagozloo et al. [7] as they noted a significant increase in CD3 and CD4 cells compared to controls. However, in another study, the percentage of CD4+ cells and T-regulatory cells (T-regs) was significantly lower in β-thalassemia major cases than in controls [8]. In our study, the difference in the CD4:CD8 ratio was not significant between cases and controls. Similarly, other studies did not reveal any significant difference in lymphocyte subsets between cases and controls [9,10].

Our study noted no significant difference in T-regs between cases and controls. However, Bozdogan et al. [11] found increased T-regs in thalassemia cases. These newly raised regulatory T cells suppress antigenic stimulation from repeated transfusions. This contrast in our results could be attributed to the stable thalassemia major cases in our study. The literature reveals that T-regs have variable effects in varied infections [12]. For a better understanding of T-regs, studies must also include active heterologous infections. A study stated that patients with thalassemia trait and thalassemia major are both in permanent immune activation status. Only thalassemia major patients are exposed to repeated antigenic stimulation, but the level of T-regs was increased in both the thalassemia trait and the major as compared to controls in their study [11]. So, the exact pathogenesis behind increased T-regs needs to be further explored.

All the cases in our study were on the oral chelator deferasirox. Despite that, there was a statistically significant difference in serum iron, ferritin, and TSAT in cases and controls (p<0.001). The iron chelators given to combat iron overload do not necessarily bring down ferritin levels [5]. It has been supported by other studies also, wherein mean serum ferritin levels were higher despite regular chelation therapy [11].

We divided our cases into iron overload and non-iron overload based on a serum ferritin level cut-off of 1000 µg/L and tried to find out if any relation exists between the iron profile and cell-mediated immunity. A significant moderately positive correlation between T-regulatory cell marker CD4+CD25+ and serum ferritin was found in 70% of cases with serum ferritin less than 1000 µg/L (p=0.031, r=0.627) when compared to cases with serum ferritin greater than 1000 µg/L. Similarly, those cases also demonstrated a moderately significant positive correlation between serum iron and absolute lymphocyte count (r=0.529, p=0.042). These findings suggest that iron can influence lymphocytes as well as T-regs. Our results were similar to those of Shokrgozar et al. [13] who found a positive correlation between T-regs and ferritin in thalassemia major patients. In contrast, increased serum ferritin levels were associated with lower CD4+ T-lymphocytes in adults with transfusion-dependent β-thalassemia in a recent study [14]. Similarly, another study documented that iron overload can affect humoral and cell-mediated immunity in patients with β-thalassemia. The researchers documented a positive correlation of ferritin with CD8, IgG, and IgA and a negative correlation with IgM, CD3, and CD4 in their patients [15]. Clinical heterogeneity, infections, or technical differences could be the possible reasons for such different results. Gray et al. [16] reported in their study that melanoma cells primarily produce a ferritin-heavy chain, which induces T-regs, resulting in the suppression of immune cells. It might suggest that ferritin has immunoregulatory properties. We also found a positive correlation between serum ferritin and T-regs in our cases. Therefore, it might be stipulated that serum ferritin influences T-regs in thalassemia major patients. Hence, as ferritin levels rise, they might suppress immunity in these patients.

The healthy controls revealed statistically significant results in our study. Serum iron and transferrin saturation showed a significant positive correlation with T-regs, indicating a direct relationship. Hence, it can be proposed that increased serum iron might induce T-reg proliferation, which might further cause immune dysfunction and increase susceptibility to infections in patients with iron overload. This finding is further supported by the significant negative correlation between serum ferritin and the percentage of CD8+ cells in controls. These results in healthy controls suggest and support the fact that serum iron and serum ferritin, even in the normal range, have substantial immunoregulatory properties.

Limitations of the study

The number of patients in our study was small. Hence, larger studies should be conducted for better statistical correlation. Some studies suggest that chelators can also affect the immune system. As a result, studies should be conducted in the future with these factors in mind.

## Conclusions

To conclude, the results of this study suggest that iron can potentially influence cell-mediated immune cells. Raised serum ferritin even in the non-overload range positively affects regulatory T cells which might suppress CD4+ and CD 8+ immune cells, increasing the risk of infections in transfusion-dependent thalassemia major patients. The significant negative correlation between serum ferritin and the percentage of CD8+ cells in healthy controls suggests that cytotoxic immunity might be deranged in iron overload scenarios. Hence, therapeutic measures in terms of better chelators or their combinations should be considered to reduce serum iron as well as ferritin stores. This study may also provide useful insights into the disease biology and therapy so that T-regulatory cell immunotherapy could be utilized in the future. The number of patients in our study was small hence larger studies should be conducted for better statistical correlation. Some studies suggest that chelators can also affect the immune status, hence future studies keeping these factors in mind should be conducted.
